# Exploring the mysterious behavior of the critically endangered South African Wolkberg Zulu butterfly (*Alaena margaritacea*) using micro-CT scanning and morphological analysis (Lycaenidae: Liptenini)

**DOI:** 10.7717/peerj.21368

**Published:** 2026-05-27

**Authors:** Pasi Sihvonen, Max Söderholm, Zowi Oudendijk, Martin H. Villet, Etienne Terblanche

**Affiliations:** 1 Finnish Museum of Natural History, University of Helsinki, Helsinki, Finland; 2 Faculty of Biological and Environmental Sciences, University of Helsinki, Helsinki, Finland; 3 Department of Zoology & Entomology, Rhodes University, Grahamstown, South Africa; 4 Research and Exhibitions Department, South African Museum, Iziko Museums of South Africa, Cape Town, South Africa; 5 Research Unit Literature and Languages in the South African Context, North-West University, Potchefstroom, South Africa

**Keywords:** Behavior, *Alaena margaritacea*, Female, Imaging, Mating plug, Micro-CT scanning, Morphology, Pheromone

## Abstract

**Background:**

The Critically Endangered endemic Wolkberg Zulu butterfly (Lycaenidae: *Alaena margaritacea* Eltringham, 1929) is known from only two colonies in the Wolkberg mountain range in north-east South Africa. An unusual behavior of this species was reported in 2020: the female climbs and criss-crosses grass blades while rubbing her abdomen against them and curling her abdomen. The function of the unusual behavior is unknown, but it might attract males by depositing a pheromone. Although long-range female sex pheromones are rare in butterflies (Papilionoidea), this conjecture is supported by a male flying to a tussock containing a female, where mating occurred directly.

**Methods:**

We used non-destructive micro-CT scanning and morphological analysis to uncover the potential abdominal and reproductive structures, including glands, that could explain the female’s behavior. We illustrate the structures on *A. margaritacea* examined using micro-CT scanning, including rendered 2-D surface photos, rendered surface videos, a video showing the entire slide stack, video showing the density differences, and focus-stacked 2-D photos.

**Results:**

We found a sclerotized area mid-ventrally on the 7th sternite, a ventral pouch-shaped opening on the 9th segment, and tiny, spherical, superficially gland-like structures inside the 7th abdominal segment. The sclerotized area on the 7th sternite may be associated with the observed behavior, and the gland-like structures may have a pheromone-producing function, but our data do not allow unambiguous confirmation of either of these explanations. However, we confirm the presence of a waxy mating plug sealing the ostium bursae, the first confirmation of this structure in a lycaenid. It corresponds to a protosphragis, extending into the posterior part of the ductus bursae and onto the ventral part of the 9th sternite. The mating plug could offer an alternative explanation for why the female rubs her abdomen against grass blades: she may be trying to remove the mating plug mechanically to allow remating. The closely related *Alaena amazoula* was also found to have a sclerotized area on the 7th sternite. We illustrate the female genitalia of *A. margaritacea* and *A. amazoula* using morphological analysis to explain their structures.

## Introduction

Lycaenidae is the second largest family of butterflies, with over 5,000 described species worldwide (Nieukerken et al., 2011). Within the subfamily Poritiinae, the tribe Liptenini is endemic to continental Africa and, unlike other Lycaenid lineages, its nearly 640 species have been recorded to feed exclusively on lichens (Boyle et al., 2023; Williams, 2006). Diet is not the only unusual feature of the Liptenini. The female of the liptenine Wolkberg Zulu (*Alaena margaritacea* Eltringham 1929) reverses typical lycaenid habits where females fly to territories established and defended by males, for instance on hilltops (see Takeuchi, 2006; Alcock & O’Neill, 1987; Shields, 1967). In contrast, females of *A. margaritacea* establish a mate-encounter site in a tussock to which males fly for mating. Indeed, female calling is unusual among diurnal Lepidoptera overall, and pheromonal glands have disappeared from Papilionoidea over evolutionary time (Boppré, 1984; Sartoi Monteys et al., 2016).

**Figure 1:**
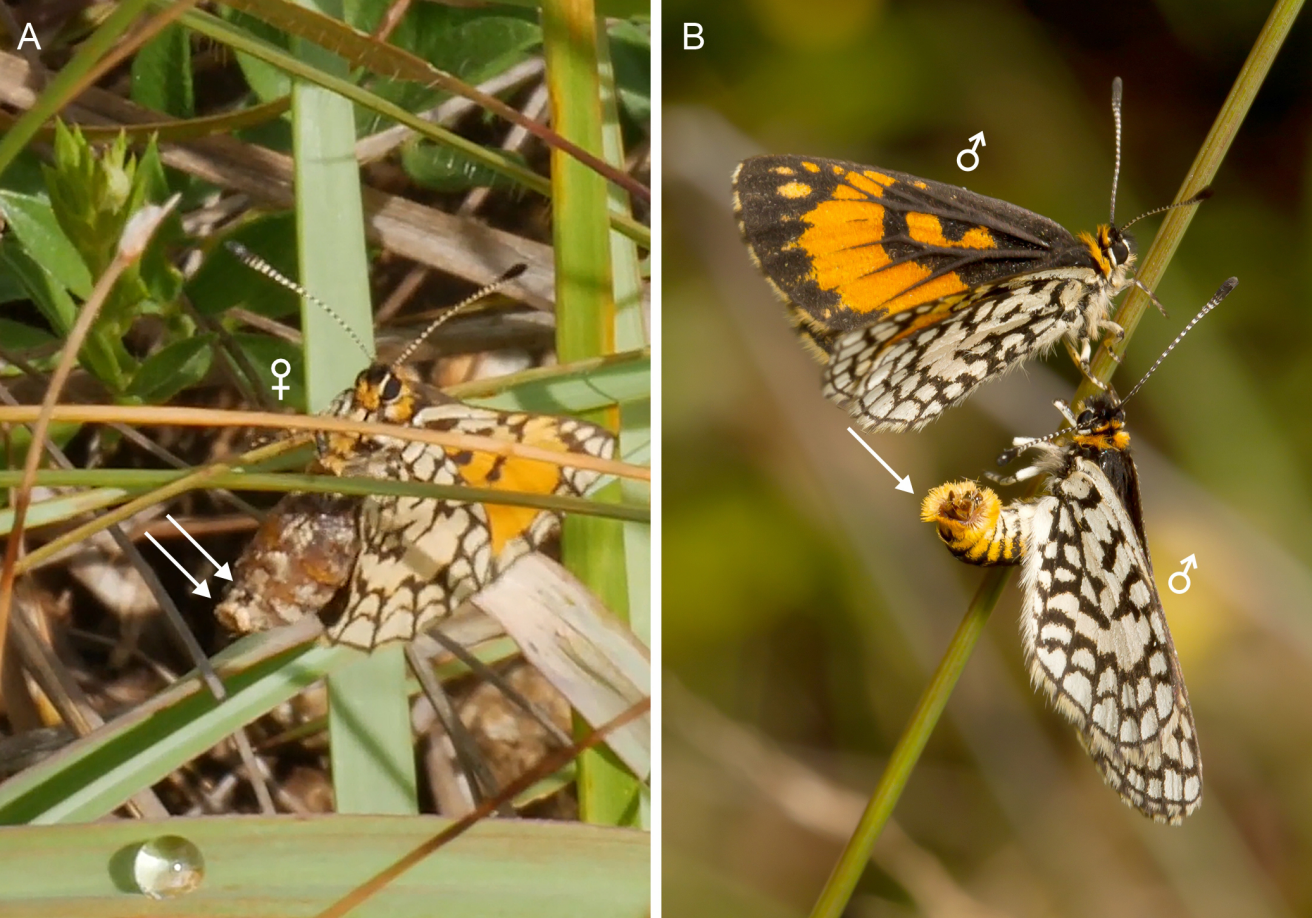
Mysterious behavior of *Alaena margaritacea*, filmed near Haenertsburg in South Africa on 1 January 2020. (A) Female walking in criss-cross fashion among the grass blades, repeatedly rubbing the ventral side of its abdomen against stems and blades, potentially indicating scent-marking behavior. The arrow on the left indicates the tip of the abdomen, which the female pokes ventrally (not visible in the photo), the arrow on the right indicates the ostium bursae. Screenshot from a video (1 min 44 s) available on Vimeo. (B) Two males among the grass, one of them ready to mate as indicated by its open valvae (arrow). We assume that the males respond to a pheromone a female secreted earlier. Photo credit: André Coetzer. The main question in our study is to find out whether the female has pheromone glands or other structures, which could explain behavior that is unusual among butterflies (Papilionoidea).

*Alaena margaritacea* (Fig. 1) is Critically Endangered and endemic (Mecenero et al., 2013; Coetzer, 2020), known from grassy slopes covered by rocks in the Haenertsburg area of Limpopo in the Wolkberg mountains in north-east South Africa (Coetzer, 2015). Only two colonies are known, both under severe threat from alien tree plantations (Coetzer, 2015; Coetzer, 2020; Mecenero et al., 2020). The species is univoltine and adults are on the wing in December and January. The mating behavior (Terblanche, 2021) and life history (Clark & Dickson, 1971; Coetzer, 2015) have been documented in detail. Mating behavior requires the presence of grass tussocks that surround loose rocks or continuous rock surfaces and the larvae feed on an unidentified dark crustose rock lichen, probably in the family Lichinaceae. Against this background, any additional information on this endangered species may prove valuable for its conservation.

In 2020, Etienne Terblanche observed a peculiar behavior in *A. margaritacea* within its natural habitat: a female was walking in criss-cross fashion among the grass blades, fanning its wings, poking the tip of its abdomen ventrally in a curled manner by means of contraction, while climbing to the grass tips repeatedly and rubbing the ventral side of its abdomen against stems and blades in a way that could potentially be scent-marking behavior involving a low-volatility or non-volatile contact pheromone. A similar behavior was observed in a different female, and in both cases the female began the behavior from the base of the tussock. Also in both cases, the female ended the behavior by perching on the top of a blade. This behavior was described in detail (Terblanche, 2020) and a video showing the behavior is available online (https://vimeo.com/416060510). Terblanche named the behavior “grass grooming,” since the insect’s legs rub the substrate quickly as it progresses in the tussock (Fig. 1A). Egg-laying behavior could be ruled out, because the behavior took place among the grass, where mating has been documented to occur (Terblanche, 2021), while females oviposit on rock surfaces where their caterpillars feed on lichens (Clark & Dickson, 1971; Coetzer, 2015). Terblanche (2020) presented four semiochemical-related hypotheses for the observed female behavior: to attract males, mark territory, repel competing females or repel feeding competitors such as ants.

Because such behavior is unusual and female butterflies (Papilionoidea) do not have pheromone glands, except occasionally close-range courtship pheromone glands (*e.g.*, Myers, 1972; Sartoi Monteys et al., 2016), we wanted to explore whether the abdomen of female *A. margaritacea*, particularly segments 6–10, contain glands that could be connected to pheromone production. For comparative purposes, we also examined the closely-related *Alaena amazoula* (Boisduval, 1847) (*e.g.*, Williams, 2006; Krüger, 2020).

Another explanation for the unusual behavior could include the recently documented mating plug or sphragis of *A. margaritacea* (Terblanche, 2021). A review focused on Papilionidae and Nymphalidae (Carvalho, Orr & Kawahara, 2017) concluded that sphragides occur in only about 1% of known butterfly species, and rejected an illustrated report of sphragides in Lycaenidae (Bryk, 1919) as misinterpreted. If Bryk’s record is indeed unreliable, *A. margaritacea* would be the only lycaenid confirmed to possess a sphragis. Current classification of mating plugs uses their structure and placement, including ornate external mating seals or sphragides; generalized internal mating plugs or protosphragides; and transitional vestigial sphragides and hemisphragides (Carvalho, Orr & Kawahara, 2017). Although the mating plug of *A. margaritacea* was examined externally using scanning electron microscopy (Terblanche, 2021), the limitations of the method left it unknown if the plug is solid or hollow; if it was folded or just brittle and cracked; if it has specific internal structures; or how deeply it extends into the female reproductive tract. Because these features may be associated with the observed female behavior, and more information is needed to understand how the lycaenid mating plug fits in the classification system devised for other Lepidoptera, we decided to also include an examination of the mating plug in our study.

Our primary method was micro-CT scanning, which allows detailed study of internal structures in a non-destructive way, supplemented by focus-stacking imaging and morphological dissections. Following this, if glands are found and their exact location becomes known, other methods like transmission electron microscopy (TEM) could then be applied to study them at cellular level.

## Materials and Methods

### Behavior

Behavior was observed at the type locality of the species outside Haenertsburg in South Africa and recorded on a Panasonic GH-4 camera. On 1 January 2020 a female’s behavior was observed. On 15 December 2021 the same behavior was observed for a second female. On 23 December 2021 a male was observed flying to a third female in a tussock, after which mating occurred directly.

The behaviors were interpreted through careful and repeated scrutiny of the recordings by Etienne Terblanche at reduced playback speeds and single frames, leading to the formulation of hypotheses about what anatomical structures would support each interpretation. For instance, the idea that females were dispersing a mating pheromone would be supported by finding glands that might produce such a pheromone. These hypotheses were then used to guide anatomical examinations of specimens using scanning electron microscopy and micro-CT scanning to seek *e.g.*, potential glands.

Behavioral video recordings are available at https://vimeo.com/416060510.

### Anatomical material

Etienne Terblanche collected the following specimens under permit from the Limpopo Department of Economic Development, Environment and Tourism: Limpopo Province Directorate: Wildlife Trade and Regulation Permit, permit number 01763.

Rhodes University scrutinized the ethical aspects of this study (RUAREC clearance number 2025-5812-9404).

Raw data index file lists the details of each image included in the study (Supplement 3).

*Alaena margaritacea* Eltringham, 1929. **Female**: SOUTH AFRICA: Limpopo — type locality near Haenertsburg — [exact site not revealed to protect the small and endangered population] — 15 January 2025 — J. E. Terblanche leg. —— specimen A — micro-CT scan #172 — Max Söderholm (coll. Terblanche, South Africa). Specimen identifier: L-ET:ET.24.1. **Female**: South Africa: Limpopo — type locality near Haenertsburg — [exact site not revealed to protect the small and endangered population] — 15 January 2025 — J. E. Terblanche leg. —— specimen B —— Pasi Sihvonen — prep. number 2969 (coll. Terblanche, South Africa). Specimen identifier: L-ET:ET.24.2. **Female**: SOUTH AFRICA: Limpopo — type locality near Haenertsburg — [exact site not revealed to protect the small and endangered population] — 1 January 2020 — J. E. Terblanche leg. —— selected structures SEM photographed. Specimen identifier: L-ET:ET.24.3. (coll. North-West University, South Africa).

*Alaena amazoula* (Boisduval, 1847). **Female**: SOUTH AFRICA: Limpopo — Bewaarkloof — -24.11169 29.89402 — 4 December 2024 — J. E. Terblanche leg. (coll. Terblanche, South Africa) —— Pasi Sihvonen — prep. number 2968. Specimen identifier: L-ET:ET-24.4.

The freshly killed specimens were kept frozen at −20 °C in South Africa, defrosted during the approximately 20-hour transport from South Africa to Finland, and frozen again at the destination.

### Morphological analyses

Abdomens and genitalia were prepared following standard methods (see, for instance, Hardwick, 1950). Wings of a defrosted specimen of each species were removed with micro scissors and glued to a rectangular plastic sheet, which was attached to an insect pin bearing labels. The abdomens and genitalia were stained with Chlorazol Black. Several structures shown in the plates were photographed *in situ* in ethanol in two to six images at different depths of focus and combined into single images using Photoshop (*v.* 2025) image-stacking software. Original images were cleaned, edited and compiled into plates using CorelDraw (*v.* 2025). Terminology for genitalia follows Klots (1970) and Sibatini (1972), and in doubtful cases, descriptive terms were used.

### Micro-CT scanning

A specimen was defrosted and stained following Niekampf et al. (2024) with some adjustments. The wings of the butterfly were removed with micro scissors (see below), while the head, thorax, and abdomen were fixed in 70% Bouin’s solution for 60 h. Afterwards, it was washed several times with 70% ethanol before being dehydrated by being bathed for an hour in each of an ascending series of ethanol concentrations (70%, 80%, 90%, 95%, 100%, 100%). The sample was stained with 1% iodine solution (prepared in absolute ethanol) for 18 h. It was later washed twice with absolute ethanol for 10 min and dried using a Leica EM CPD300 Critical Point Dryer (Leica Microsystems GmbH, Austria); provided by EMBI Bioimaging, University of Helsinki, Finland.

The specimen was imaged using a Nikon XT H 225 micro-CT scanner. It was glued on a piece of cardboard to stabilize it, allow easy handling and enable precise positioning in the scanner. Imaging was performed using a molybdenum setting on the multi-metal target with the following parameters: 80 kV beam energy, 84 µA beam current,1.42 s exposure time, 4.476 projections, with a two-frame averaging per projection. The total acquisition time was 3 h 31 min 14 s.

The volume was reconstructed from the projection images using Nikon CT Pro 3D (Version XT 6.9.1) and had an isotropic voxel size of 3 µm. Inspection, analysis, and visualization, including the rendering of images and videos, were performed in VGSTUDIO MAX 2024.3.

Micro-CT scan data for *Alaena margaritacea* (specimen L-ET:ET.24.1) are available *via* MorphoSource as individual media records at https://www.morphosource.org/projects/000799152. These include volumetric image series (Supplement 3 https://www.morphosource.org/concern/media/000803676?locale=en) and rendered videos (Supplement 1 https://www.morphosource.org/concern/media/000803682?locale=en, Supplement 2 https://www.morphosource.org/concern/media/000803679?locale=en). A raw data index linking each figure panel to its underlying files is provided in File S4 (xlsx).

## Results

A video recording available online (https://vimeo.com/416060510) shows a female moving on grass blades and fanning its wings, with the wings held primarily in an open, almost coplanar posture and the forewings pulled well forward to expose most of the bright yellow underside of the forewing and upper side of the hind wing. The females observed appeared to rub their 7th and 8th abdominal sternites on the grass blades on which they were clambering and curled and poked their abdomens ventrally as though dispersing a pheromone. Observations were therefore focused towards the sternites and terminalia of the female. The posterior segments of the female abdomen are visualized in the supplementary video files (Supplements 1 and 2).

**Figure 2:**
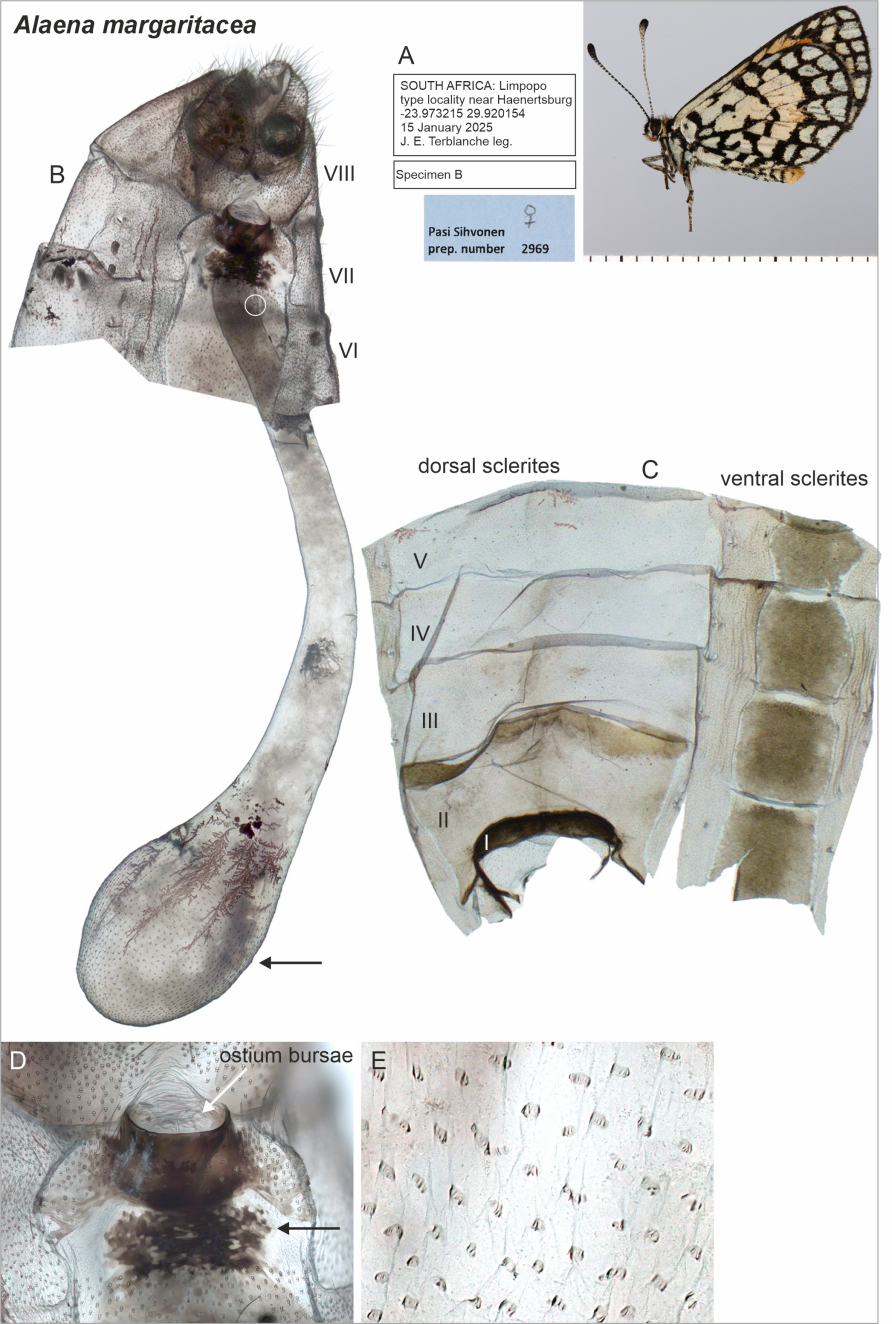
Selected female structures of *Alaena margaritacea*. (A) Adult, lateral view. (B) Genitalia with segments indicated. The point of origin of the ductus seminalis is indicated with a white circle. The black arrow indicates the area enlarged in (E). (C) Descaled abdomen with segments indicated. (D) Ostium bursae and the sclerotization mid-ventrally (indicated by the black arrow) on the 7th segment. (E) Detail of signa.

**Figure 3:**
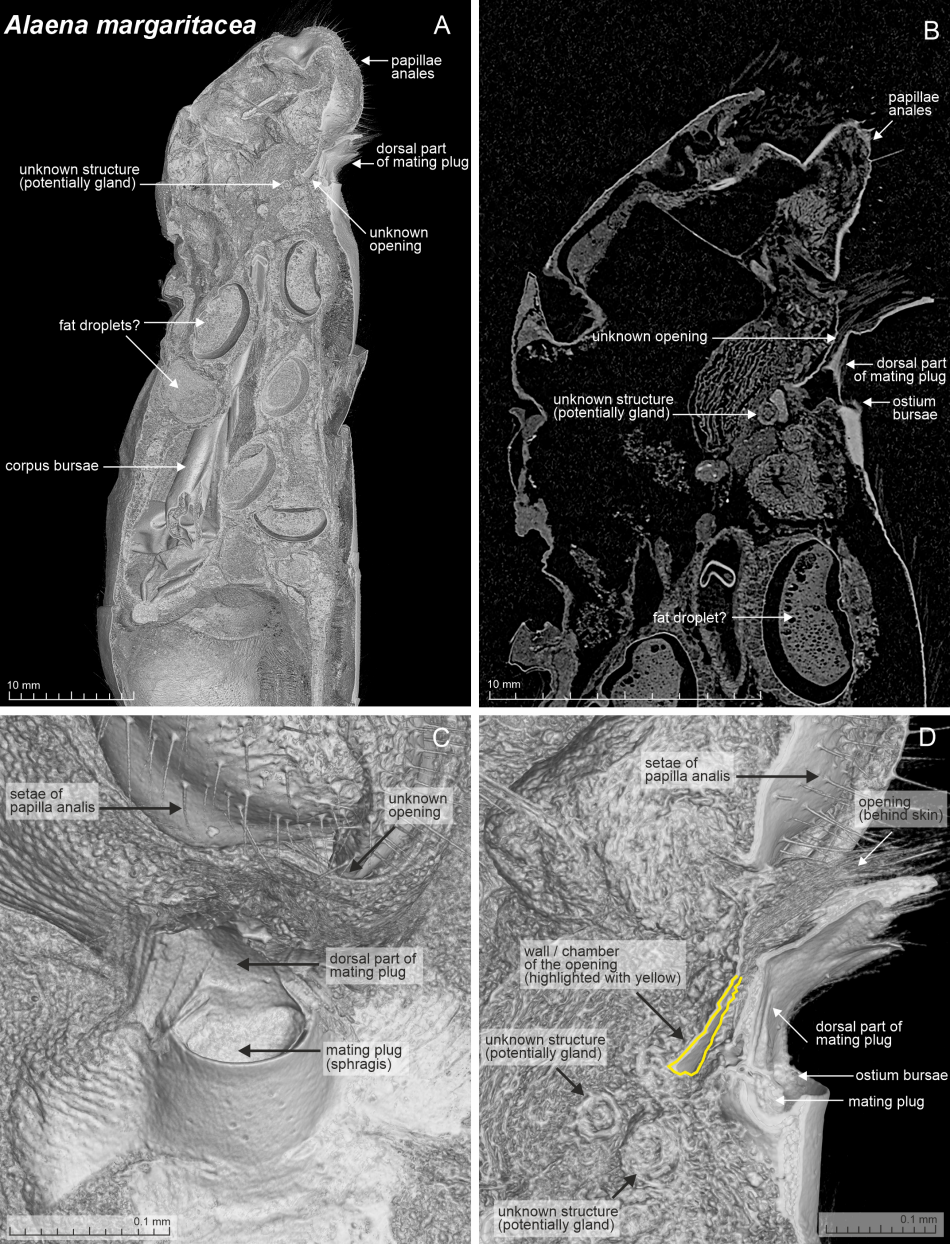
Selected micro-CT scan female structures of *Alaena margaritacea*. (A) Volume rendering of the posterior part of the female abdomen in lateral view, with a cut-through along the midline, revealing the internal structures. (B) Single grayscale slice image of the posterior part of the female abdomen in lateral view, taken around the midline. (C) Volume rendering of the ostium bursae and adjacent structures from a ventral view, showing the external surface morphology. (D) Zoomed-in volume rendering from panel A, focusing on the area near the ostium bursae. Two potential gland-like structures inside the 7th segment are highlighted. These spherical structures may have a pheromone-producing function, being candidates for more detailed transmission electron microscopy (TEM) examination.

**Figure 4:**
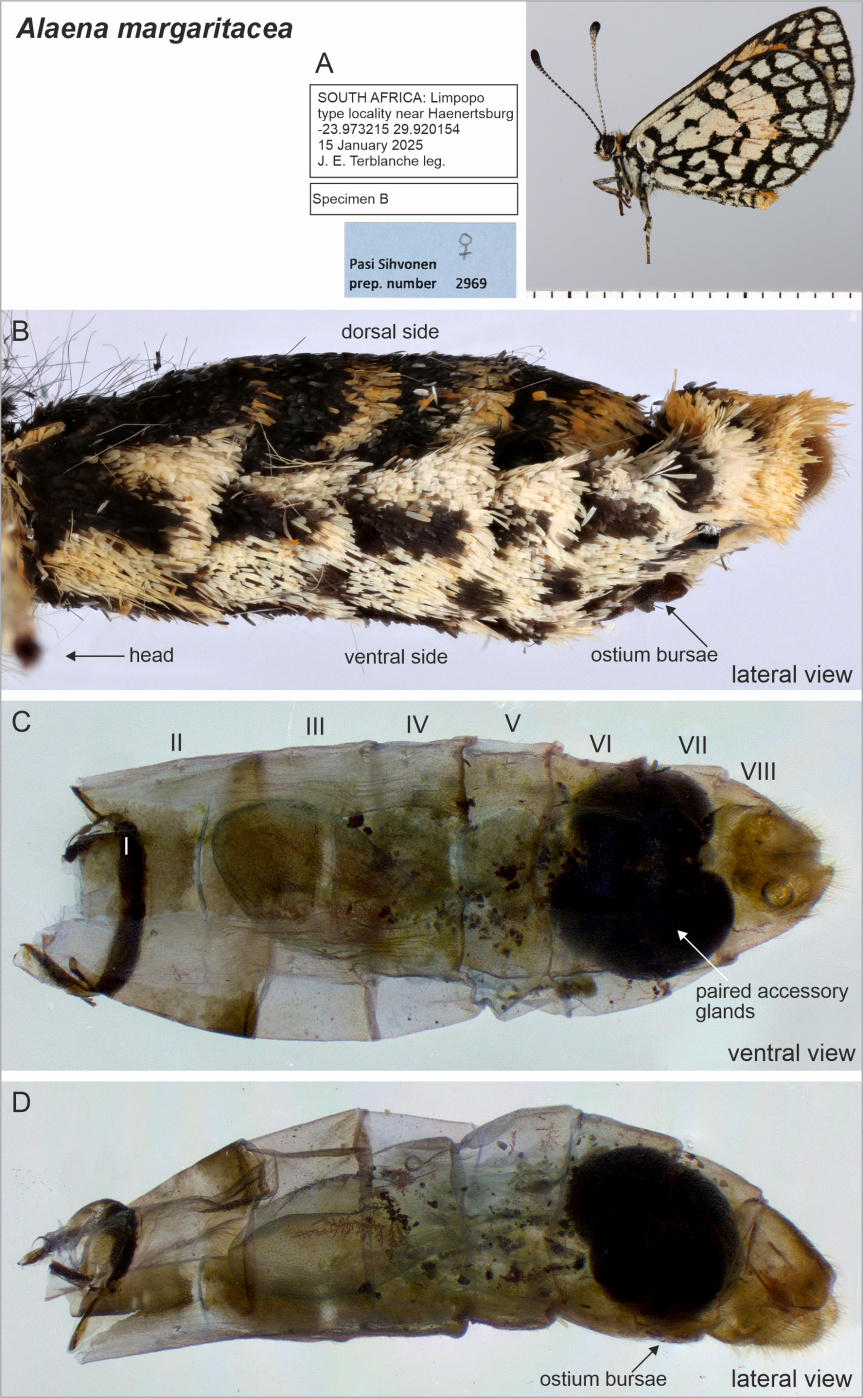
Selected female structures of *Alaena margaritacea*. (A) Adult, lateral view. (B) Abdomen, lateral view with ostium bursae indicated. (C) Descaled abdomen, ventral view with segments and massive paired accessory glands indicated. (D) Descaled abdomen, lateral view.

Beneath the scales, there was a sclerotized area mid-ventrally on the 7th sternite in *A. margaritacea* (Fig. 2D), a ventral pouch-shaped opening on the 9th segment, and tiny spherical structures inside the abdomen superficially resembling gland-like structures (Fig. 3). The sclerotized area on the 7th sternite was distinctly darker than the rest of the membranous segment, and might be associated with the observed grooming behavior, while the spherical gland-like structures inside the 7th segment may have a pheromone-producing function, but our data do not allow us to confirm either of these functions. The genitalia have large, paired accessory glands that fill up most of the 6th and 7th segments (Fig. 4). These glands contain a dark, almost black substance. The scales around the ostium bursae were not different from the scales found elsewhere on the abdomen (Fig. 4). The posterior segments of the female abdomen are visualized in the supplementary video files (Files S1 and S2).

The female genitalia of the closely related *A. margaritacea* and *A. amazoula* have not previously been described or illustrated. They allow safe species-level determination (Figs. 2 and 4) and their diagnostic features include for instance the microsigna in their corpus bursae (minute signa evenly distributed in anteriorly in *A. margaritacea*—in a narrow belt in *A. amazoula*); proximal width of the ductus bursae (wide in *A. margaritacea*—narrow in *A. amazoula*); shape of the ostium bursae (sclerotized, bowl-shaped in *A. margaritacea*—membranous, laterally elongated in *A. amazoula*); and the shape of the anterior part of the corpus bursae (round in *A. margaritacea*—elongated in *A. amazoula*).

**Figure 5:**
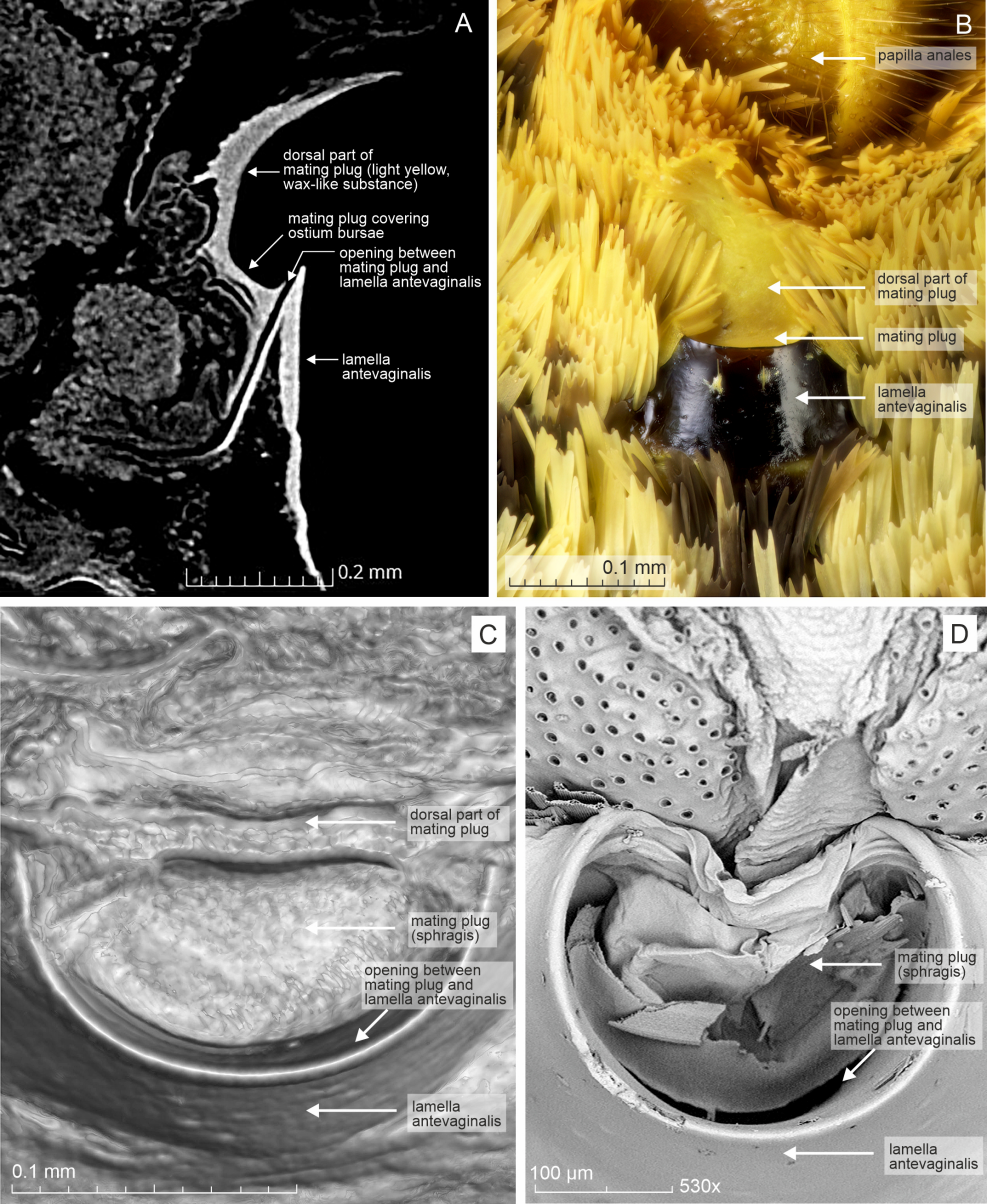
Selected female structures of *Alaena margaritacea*. (A) Single micro-CT grayscale slice image of the posterior part of the female abdomen in lateral view, taken around the midline. The image shows the mating plug, which fills the posterior part of the ductus bursae and the ostium bursae, and extends into the lamella postvaginalis. (B) Ostium bursae and the adjacent structures in ventral view. The mating plug is a pale yellow, wax-like structure that covers the ostium bursae and extends into the lamella postvaginalis. (C) Single micro-CT grayscale slice image of the ostium bursae in view directly from above. Note the narrow opening between the lamella antevaginalis and the mating plug, which is also visible in the micro-CT scan in Figure A. (D) SEM photograph of the ostium bursae, showing flaky and layered mating plug. The difference between the mating plugs is likely an artefact caused by drying of the material. Photo credit: Willie Landman.

The mating plug (sphragis) was a pale yellow, wax-like substance covering the ostium bursae entirely, except for the anterior margin (Fig. 5). The narrow, curved opening on the anterior margin could be an artefact resulting from shrinkage of the mating plug during critical point drying. The mating plug was solid, filling the sclerotised part of the ductus bursae and extending externally into the lamella postvaginalis. The classification of this lycaenid plug cannot readily be interpreted within the classification developed by Carvalho, Orr & Kawahara (2017), probably because their study excluded the Lycaenidae.

**Figure 6:**
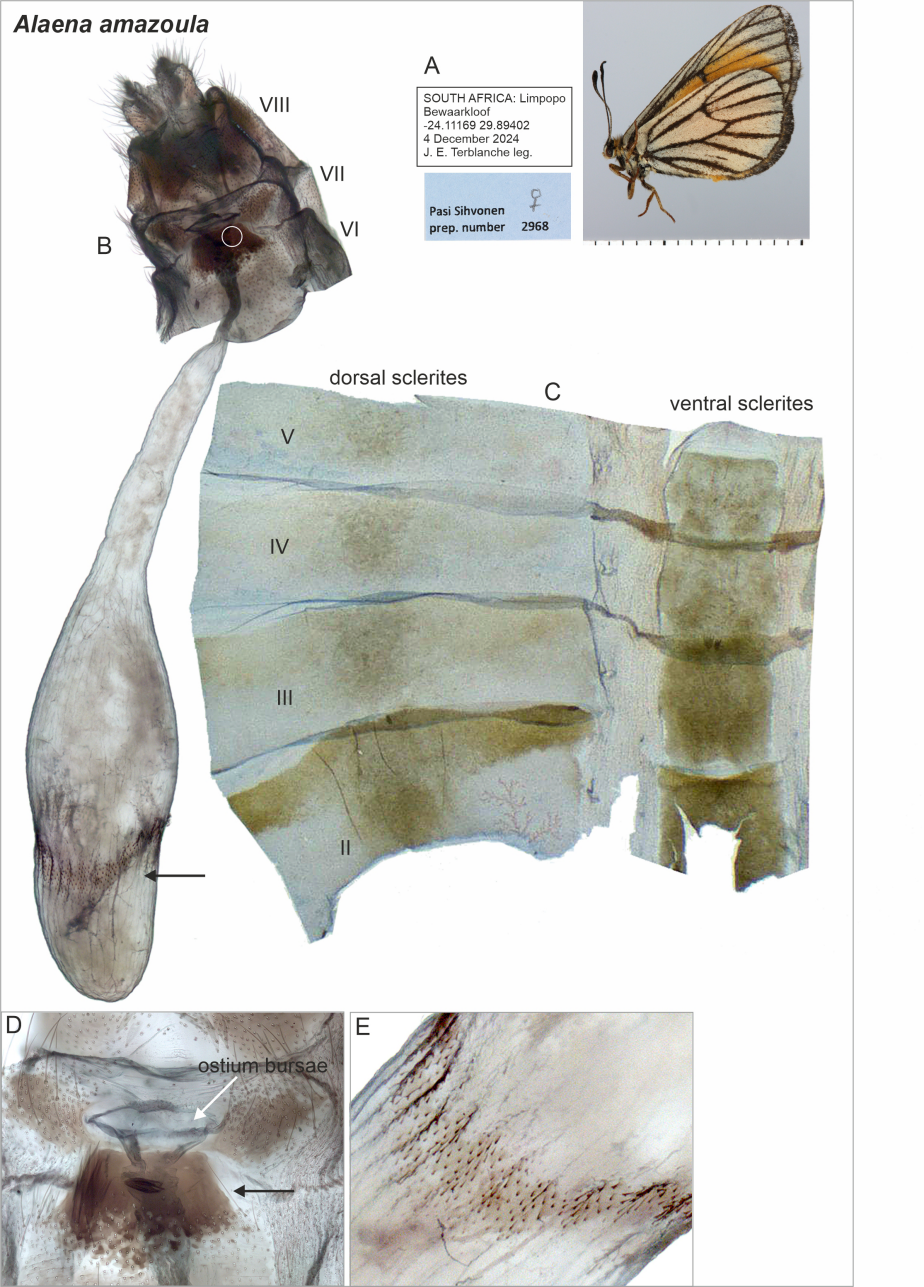
Selected female structures of *Alaena amazoula*. (A) Adult, lateral view. (B) Genitalia with segments indicated. The point of origin of the ductus seminalis is indicated with a white circle. The black arrow indicates the area enlarged in (E). (C) Descaled abdomen with segments indicated. (D) Ostium bursae (white arrow) and the sclerotization mid-ventrally (black arrow) on the 7th segment. (E) Detail of signa.

*Alaena amazoula* was found also to have a sclerotized area mid-ventrally on the 7th sternite (Fig. 6). We did not observe large accessory glands. *Alaena amazoula* was not examined using micro-CT scanning.

To conclude, we did not find structures that could be unambiguously identified as pheromone-producing.

## Discussion

Generally, male butterflies in Papilionoidea use vision to detect conspecific females. Female butterflies, in turn, lack long-range sex pheromone glands to attract males (Boppré, 1984; Sartoi Monteys et al., 2016), although females of some species, for instance in Nymphalidae: Danainae, release close-range pheromones to signal readiness to mate (Myers, 1972). On the other hand, males of *Pieris* may mark their mates with scents that put off subsequent suitors (Mozuraitis et al., 2019). Against this background, our visual exploration to find potential pheromone-producing glands in the posterior abdominal segments (A6–A10) in *A. margaritacea* entailed an exploratory approach. Using both micro-CT scanning and morphological dissections, we could not detect internal or external structures that could be unambiguously identified as pheromone glands, such as eversible membranes, porous areas, or specialized abdominal scales that could broadcast such a pheromone.

Using the micro-CT approach, however, we identified small spherical-glandular structures in segment A7, situated near the ostium bursae and the mid-ventral sclerotization on sternite S7 (Fig. 3). These structures are potential gland candidates, and they should be investigated in detail, for instance to learn if there are ducts leading to the intersegmental membrane between sternites S8–S9 or another intersegmental membrane. The spherical glandular appearance and slightly elongated shape allow us to conclude these are not eggs. Potential methods to be employed for further exploration of the matter could include transmission electron microscopy (TEM) to study the structures on the cellular level.

Pheromone glands and other scent-producing structures are common in female moths, often located in the intersegmental membrane between segments A8 and A9, or in the body cavity beneath the 7th and 8th abdominal tergites (Percy-Cunningham & MacDonald, 1987; Suwanjarat & Witethom, 1995; Ma et al., 2017; Zohry, 2013). Our initial finding of potential glands inside the 7th segment aligns with the position of these structures in moths.

The sclerotization on the mid-ventral part of sternite S7 in both *A. margaritacea* and *A. amazoula* indicates that the region is reinforced for physical contact with some other structure. This interaction could be centred on contact with grass blades, but it could also be mating-related, that is, it could be a secondary sexual structure interacting with the male genitalia or abdomen during copulation. Such correlating sclerotizations are known for instance in a Euro-Siberian geometrid moth *Scopula immorata* (Linnaeus, 1758), where the sclerotized and brush-like structures on sternite S8 of the male were found to brush against the sclerotized lamella antevaginalis of the female during copulation. The interaction of these structures suggests that the male organs are involved in the tactile sexual stimulation of the female (Sihvonen, 2007).

We confirm the earlier finding (Terblanche, 2021) that the male of *A. margaritacea* deposits a mating plug into the female genitalia during copulation (Fig. 5). In the present study, the portion of the protosphragis sealing the ostium bursae is solid and relatively smooth, whereas the structure illustrated previously appeared flaky and layered (Terblanche, 2021). We assume the solid condition is the natural state and the flaky and layered state is likely an artefact caused by drying of the material.

Sphragid or protosphragid mating plugs are now known from the Papilionoidea families Papilionidae and Nymphalidae (Carvalho, Orr & Kawahara, 2017), Pieridae (Orr, 1988), and Lycaenidae (Terblanche, 2021, this study). The Category 1 sphragis—into which the structure of *A. margaritacea* best fits—is relatively rare; only 9% of the species examined by Carvalho, Orr & Kawahara (2017) belong to this category, and none of these were Lycaenids. It is not clear if this is a bias caused by the relative difficulty of detecting mating plugs that are largely internal. Most sphragides are solid (73% in the dataset of Carvalho, Orr & Kawahara, 2017), as is the case here. We anticipate that additional sphragis-bearing species will be discovered, because small internal structures, as in *A. margaritacea* and recently examined *Pteronymia* butterflies (Nymphalidae: Carvalho, Mota & Kawahara, 2019), are easily overlooked.

The small, semi-internal plugs that are common among ditrysians are not visible externally (Matsumoto & Orr, 2021; Orr, 1995). The plug in *A. margaritacea*, which is clearly visible externally (Fig. 5B), might represent either a protosphragis or a hemisphragis. Alternatively, it may embody a new type, and it does belong among plugs of the rare intermediary type (see Carvalho, Orr & Kawahara, 2017).

Sphragis-bearing species share several morphological and behavioral traits, even across phylogenetically distant taxa. One such trait is the externalization of the female sexual opening (ostium bursae), whereas in most Ditrysia—which generally lack a sphragis—the ostium bursae is situated within a pouch-like sterigma (Carvalho, Mota & Kawahara, 2019). *Alaena margaritacea* conforms to this pattern too: its sclerotized ostium bursae is bowl-shaped and protruding, supporting the idea of parallel evolution of similar structures in distantly-related sphragis-bearing species (see Matsumoto & Orr, 2021); these adaptations involve an evolutionary response from females that resists the attachment of sphragides.

Furthermore, it is important to determine whether *A. margaritacea* is mono- or polyandrous and how this condition relates to the presence of small signa in the corpus bursae. Comparative evidence suggests that signa originally evolved to rupture spermatophore envelopes in polyandrous species, and that the evolution of monandry is associated with the reduction or loss of signa (Sánchez, Hernández-Baños & Cordero, 2011; Xochipiltecatl, Cordero & Baixeras, 2022). The presence of small signa in *A. margaritacea* (Fig. 2) therefore supports the hypothesis of polyandry, whereas the sealing of the ostium bursae by males with a mating plug renders females effectively monandrous. However, the comparatively smaller (*i.e.,* reduced) signa in *A. margaritacea* than in the closely related *A. amazoula* (Figs. 2, 4) may indicate that *A. margaritacea* is evolving toward monandry. Such a mechanistic explanation may be too simple because a plug does not eradicate polyandry (Orr, 1995; Orr, 1988) and the issue of polyandry deserves further research.

**Figure 7:**
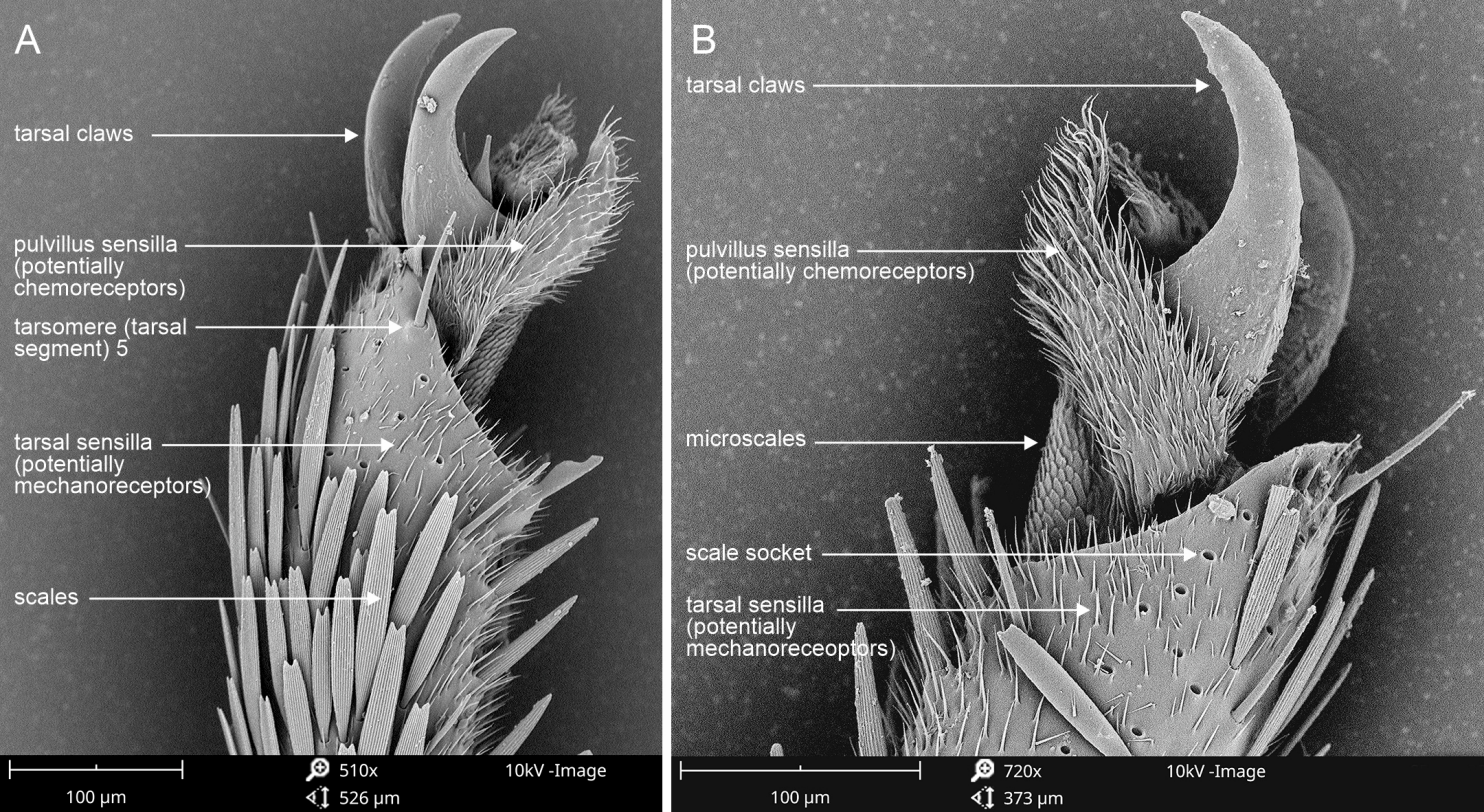
SEM photographs of the apical tarsal segment, setose pulvilli and pretarsal claws of *Alaena margaritacea* female. The setose pulvilli may involve “tasting” for the success of pheromone deposition on grass blades, supported by the observation that the female goes over the same part of the blade more than once while grooming. The pulvilli are pad-like and pale yellow, unlike other structures, which are sclerotised and dark brown. (A) External view. (B) Internal view. Photo credits: Willie Landman.

The potential function of the female’s grass-grooming behavior needs to be understood in the context of the mating behavior of *A. margaritacea*. “Grooming” is a term for quick legwork on substrata (Elmquist et al., 2018). As it moves within the tussock, poking its abdomen and wafting its wings, the female continuously grooms the blade surfaces with its legs, at great speed. This behavior may involve “tasting” for the success of pheromone deposition or the presence of pheromones from other females. That the female goes over the same part of the blade more than once while grooming and that she has sensilla around her claws (Fig. 7) support this idea.

Males have been reported to persistently perch on grass blades or stems at distances of between 5–15 cm from the grass tips, whereas females perch at the top of the blade once they have finished the peculiar behavior described here (Terblanche, 2021). A male was observed flying to a female on a blade in her tussock, and mating ensued directly after one or two quick twirls around the female. A picture taken by André Coetzer moreover shows a flying male approaching a perching male on a grass blade (Fig. 1B). The perching male’s claspers are opened, potentially indicating that the male insect is responding to a pheromone that a female has secreted (Coetzer, pers. comm., 2021), and that the male does not only respond to a visual cue, if it responds to such a cue at all.

The presence of a mating plug offers another possible explanation for the observed female behavior. The female may be attempting to remove at least the external portion of the plug mechanically, which could enable subsequent remating (polyandry). Further, hypothetically the mating plug could make contact with a potentially digestive or absorptive epithelium at the end of the ductus bursae and could provide additional nutrients for embryo development (*e.g.*, Arnqvist & Nilsson, 2000; Wong, Meunier & Kölliker, 2013), which could be an interesting topic for future research.

The function or functions of the massive paired accessory glands in *A. margaritacea* are unknown (Fig. 4). Accessory glands usually secrete large volumes of substances with functions like egg adhesion to a surface or egg protection through secretions that form protective coatings around eggs (Bueno da Silva & Costa-Leonardo, 2025). *Alaena margaritacea* lays clutches of 1–5 eggs and sticks them to rock surfaces without coating them (Clark & Dickson, 1971; Coetzer, 2015). The closely related *A. amazoula* did not have large accessory glands, and in fact they were not even noticed during the dissection. This initial finding needs more research.

## Conclusions

An unusual behavior of the Critically Endangered South African butterfly *Alaena margaritacea* was observed in 2020: the female climbs and criss-crosses grass blades while rubbing her abdomen against grass blades and curling her abdomen. The function of this unusual behavior is unknown, but hypothetically the behavior might serve to attract males by depositing a pheromone. We used non-destructive micro-CT scanning and morphological analysis to uncover the potential abdominal structures that could explain the female’s behavior. We found a sclerotized area mid-ventrally on the 7th sternite, a ventral pouch-shaped opening on the 9th sternite, and tiny spherical structures inside the abdomen on the 7th segment superficially resembling gland-like structures. Further, we confirm the presence of a waxy mating plug, the first documented Lycaenid species to definitively have this structure. All mentioned structures could explain the observed behavior, but our data do not allow unambiguous confirmation of any of them.

Our findings further highlight the unique biology, including mating behavior, and morphology of the Critically Endangered *A. margaritacea* butterfly and underscore how limited our knowledge of the species remains. The confirmation of a mating plug also suggests the presence of an intersexual arms race, which is noteworthy from an evolutionary perspective. Together, these insights reinforce the need to continue conserving the habitats on which this rare species depends.

##  Supplemental Information

10.7717/peerj.21368/supp-1 Supplemental Information 1 Raw data The details for each image included in the study.

## Additional Information and Declarations

### Field Study Permissions

The following information was supplied relating to field study approvals (*i.e.*, approving body and any reference numbers): Rhodes University scrutinized the ethical aspects of this study (RUAREC clearance number 2025-5812-9404). Limpopo Department of Economic Development, Environment and Tourism: Limpopo Province Directorate: Wildlife Trade and Regulation issued the collecting permit (number 01763).

### Data Availability

The following information was supplied regarding data availability: The volume rendering videos of *Alaena margaritacea*, showing the posterior part of the female abdomen in lateral view with a cut-through moving in the lateral direction (000803682) and showing the posterior portion of the female abdomen visualized in X-ray mode with false-colored densities (000803679); and the volumetric image series of *Alaena margaritacea*, showing the posterior portion of the female abdomen (000803676) are available at Morphosource: - DOI: 10.17602/M2/M803682, ID:000803682, https://www.morphosource.org/concern/media/000803682?locale=en
- DOI: 10.17602/M2/M803679. ID: 000803679, https://www.morphosource.org/concern/media/000803679?locale=en
- DOI: 10.17602/M2/M803676, ID: 000803676, https://www.morphosource.org/concern/media/000803676?locale=en
The raw data is available in the Supplemental File.
